# Network pharmacology integrated with molecular docking and molecular dynamics simulations to explore the mechanism of Tongxie Yaofang in the treatment of ulcerative colitis

**DOI:** 10.1097/MD.0000000000039569

**Published:** 2024-09-06

**Authors:** Lili Tang, Yuedong Liu, Hongwu Tao, Wenzhe Feng, Cong Ren

**Affiliations:** a Liaoning University of Traditional Chinese Medicine, Shenyang, China; b The Third Affiliated Hospital of Liaoning University of Traditional Chinese Medicine, Shenyang, China; c Affiliated Hospital of Shaanxi University of Chinese Medicine, Shenyang, China.

**Keywords:** meta-analysis, molecular mechanism, network pharmacology, Tongxie Yaofang, ulcerative colitis

## Abstract

Tongxie Yaofang (TXYF), a classical traditional Chinese medicine, is commonly used in China to treat ulcerative colitis (UC). The aim of this study was to integrate network pharmacology with molecular docking and molecular dynamics simulations to explore the mechanism of Tongxie Yaofang in the treatment of UC. The traditional Chinese medicine systems pharmacology database was used to retrieve the relevant chemical compositions of the herbs contained in TXYF. The DisGeNET, GeneCards, Online Mendelian Inheritance in Man, and Therapeutic Target Database databases were used to retrieve UC-related targets. To construct protein–protein interaction networks and screen for key targets, gene ontology and Kyoto Encyclopedia of Genes and Genomes analyses of the key targets of TXYF in the treatment of UC were performed using R 4.3.2 software. AutoDock Tools 1.5.7 was used for molecular docking. Molecular dynamics simulations of protein complexes and complexes of proteins with small-molecule ligands and eutectic ligands were carried out with Gromacs 2022 software. Network pharmacology analysis revealed that TXYF could act on UC through multiple targets and pathways. It may exert therapeutic effects mainly through the AGE/RAGE, TOLL, JAK/STAT, and Th17 signaling pathways. The possible targets of TXYF in the treatment of UC could be AKT1, BCL2, EGFR, HMOX1, HSP90AA1, and TGFβ1. Molecular docking analysis revealed that AKT1 had the highest binding energy (‐10.55 kcal/mol). Molecular dynamics simulations revealed that the complexes formed by the AKT1 protein and the chemical compounds MOL001910 and MOL00035 had good stability and high binding strength. AKT1 may be the most critical target of TXYF in treating UC, and the key chemical components of TXYF in treating UC may include β-sitosterol (MOL000358) and 11alpha,12alpha-epoxy-3beta-23-dihydroxy-30-norolean-20-en-28,12beta-olide (MOL00 1910). This study revealed that TXYF may exert therapeutic effects on UC through multiple targets, multiple biological functions, and multiple signaling pathways. This study provides a new insight into the pharmacological mechanism of TXYF in treating UC.

## 1. Introduction

Ulcerative colitis (UC) is an intestinal inflammatory disease that is clinically characterized by mucous, bloody stools, and abdominal pain.^[1,2]^ UC has become a global disease, and as one of the most difficult-to-cure diseases worldwide, UC has received widespread attention.^[3,4]^ This disease may occur at any age, with a high incidence rate among young adults.^[5]^

Modern medicine mainly treats UC with drugs such as aminosalicylic acid, corticosteroids, immunosuppressants, and biologics. Although the initial treatment effects are significant, they are associated with challenges, such as a high recurrence rate after discontinuation, multiple adverse reactions, hormone dependence, and high costs, rendering it difficult for some patients to accept treatment.^[6]^

Traditional Chinese medicine (TCM) has advantages such as a low cost, regulation of the intestinal microbiota, and low drug resistance, and many classical TCM formulas have good clinical effectiveness.^[7]^ Therefore, exploring the ideas and methods of TCM for the treatment of UC is highly valuable for leveraging the advantages of TCM. Tongxie Yaofang (TXYF) is a classical TCM prescription for treating diarrhea caused by liver depression and spleen deficiency. TXYF originates from the book “Dan Brook Heart Law” and consists of 4 medicines, namely, Saposhnikoviae Radix, Paeoniae Radix Alba, Rhizoma Atractylodis Macrocephalae, and Citri Reticulatae Pericarpium. The drug composition is shown in Table 1. TXYF soothes the liver, regulates the spleen, promotes yang, and stops diarrhea. Currently, TXYF is used to treat diarrhea-related diseases such as UC and irritable bowel syndrome (IBS) and has significant therapeutic effects.^[8,9]^

**Table 1:** Composition of Tong Xie Yao Fang.

Chinese name	Herbal name	Latin name	Family	Genus	Part used
Baishao	Paeoniae Radix Alba	*Cynanchum otophyllum* Schneid.	Asclepiadaceae	Cynanchum Linn.	Root
Baizhu	Rhizoma Atractylodis Macrocephalae	*Atractylodes macrocephala* Koidz.	Compositae	Atractylodes	Root
Chenpi	Citri Reticulatae Pericarpium	Citrus reticulata Blanto	Rutaceae	Citrus L.	Pericarp
Fangfeng	Saposhnikoviae Radix	*Saposhnikovia divaricata* (Turcz.) Schischk.	Apiaceae	Saposhnikovia Schischk	Root

Currently, relatively few studies have investigated the mechanism and pharmacodynamics of TXYF in treating UC, and the underlying mechanism is not yet clear. Network pharmacology can be used to predict the chemical ingredients of TCM formulas, as well as the molecular mechanisms of targets, pathways, and other effects on related diseases.^[10]^ Molecular docking can be used to evaluate the binding strength between key targets and chemical components of TCMs.^[11]^ Molecular dynamics simulations can compensate for the shortcomings of molecular docking, which is semiflexible and cannot consider the flexibility of the protein structure; thus, molecular dynamics simulations further estimate the degree and stability of the binding between pharmaceutical chemical ingredients and proteins.^[12]^ Therefore, this study integrated network pharmacology with molecular docking and molecular dynamics simulations. A “drug-target-disease” analysis model was used to explore the molecular mechanism of the multicomponent, multipathway, and multitarget effects of TXYF on UC and to predict potential chemical ingredients, key targets and the binding stability between key targets and TXYF ligands in treating UC, thus providing a reference for the treatment of UC.

## 2. Materials and methods

**Figure 1.**
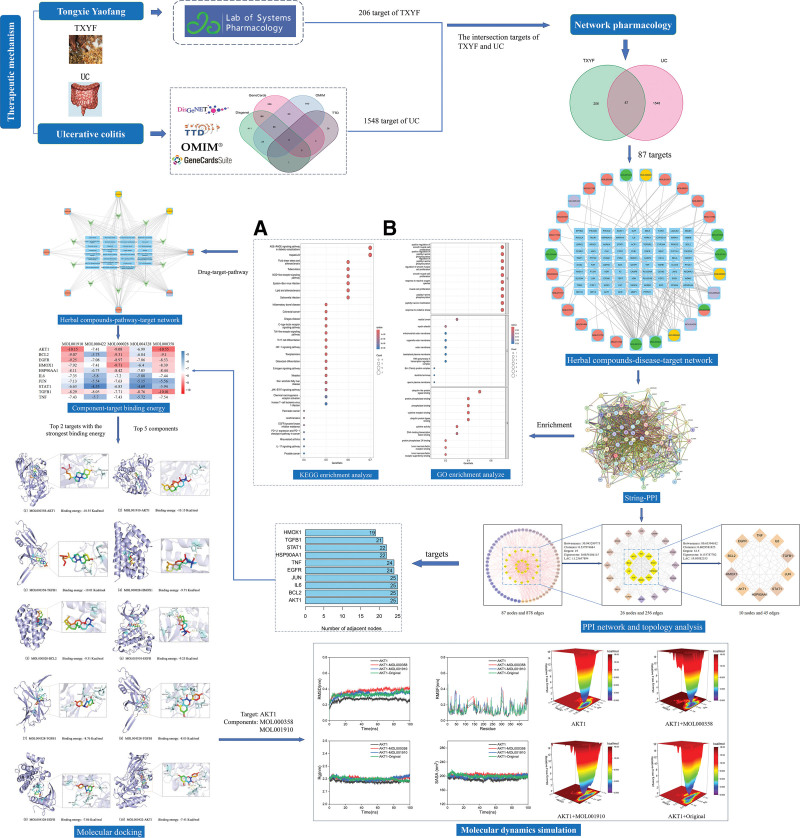
General flow chart of the study.

For a flow chart of this study, please refer to Figure 1.

### 2.1. Network pharmacology and molecular dynamics simulations

#### 2.1.1. Screening of the chemical ingredients and targets of TXYF

The traditional Chinese medicine systems pharmacology database (TCMSP) database (https://old.tcmsp-e.com/tcmsp.php) was used to retrieve the relevant chemical components of the herbs contained in TXYF, and the pharmacokinetic parameter filtering criteria were oral bioavailability ≥30% and a drug likeness ≥0.18. SwissTargetPrediction (http://www.swisstargetprediction.ch/) was carried out on the chemical ingredients of relevant targets not recorded in the TCMSP database for target prediction, and the top 15 targets were screened.

#### 2.1.2. Acquisition and screening of UC-related targets

The DisGeNET (http://www.disgenet.org/), GeneCards (https://www.genecards.org/), Online Mendelian Inheritance in Man (https://omim.org/), and Therapeutic Target Database (https://db.idrblab.net/ttd/) databases were used to retrieve UC-related targets. The intersecting targets of TXYF and UC were obtained and imported into Cytoscape 3.9.1 software to construct a drug–disease–target visualization network diagram, and the Venn 2.1.0 platform was used to construct a Venn diagram of the intersecting targets.

#### 2.1.3. Construction of protein–protein interaction (PPI) networks and screening of key targets

We used the STRING database (https://www.String-db.org/) to analyze the intersecting targets of TXYF and UC under the conditions of: “*Homo sapiens*” and “highest confidence 0.9” and to construct a PPI network. We then imported the obtained data into the Cytoscape 3.9.1 software for visual analysis, and targets with values greater than the median values for the degree, closeness, betweenness, eigenvector, and local average connectivity were selected as key targets.

#### 2.1.4. Gene ontology (GO) and Kyoto Encyclopedia of Genes and Genomes (KEGG) enrichment analyses

GO and KEGG analyses of the key targets of TXYF in the treatment of UC were performed via R 4.3.2 software, and the “clusterProfiler” program was used for enrichment analysis. The results were plotted via “enrichplot” and “ggplot2.”

### 2.2. Molecular docking

Key targets that were selected from the PPI network were identified as protein receptors, and the relevant molecular structures were obtained from the RCSB PDB database (https://www.rcsb.org/). The active drug ingredients with the strongest correlation with the key targets were screened as small-molecule ligands based on their degree value. The structures of the small-molecule ligands were obtained from the TCMSP database. Proteins were hydrotreated with AutoDock Tools 1.5.7; small molecules were also hydrotreated, and reversible bonds were determined. Grid plates were used to set the molecular docking parameters and docking range parameters (center: 12.3, ‐16.2, ‐11.2; size: 18.75 × 18.0 × 18.0), the docking mode was semiflexible, the docking accuracy was exhaustiveness = 25, and the docking algorithm was the Lamarckian genetic algorithm. AutoDock Vina software 1.1.2 was used to perform molecular docking. The binding strengths of key TXYF targets and chemical ingredients were predicted, and the binding energies of various ligands and receptors were determined. R Studio was used to construct binding energy heatmaps, and PyMOL 4.6.0 software was used to construct molecular docking diagrams.

### 2.3. Molecular dynamics simulation

To verify the binding ability and stability of drugs for targeting, we carried out molecular dynamics simulations on the basis of molecular docking. The specific method was as follows: the eutectic ligand in the related protein was redocked with the original protein, and the root mean square deviation (RMSD) values of the eutectic ligand before and after molecular docking were compared via the “align” command of the PyMOL software. An RMSD of <2 indicated that the methodology was verified. The first 2 molecular docking results with the strongest binding energies were selected and subjected to methodological verification, and molecular dynamics simulations of the complexes of proteins and proteins with small-molecule ligands and eutectic ligands were carried out with the Gromacs 2022 software. The protein force field was Amber14sb, the ligand force field was Gaff2, the solvent was added to the protein–ligand system by the SPC/E water model, a water box with a periodic boundary of 1.2 nm was established, and the charges in the system were kept in balance by adding sodium and chloride ions. Before the formal kinetic simulation, we used a conjugate gradient algorithm to make the energy of the complex reach 50,000 steps, and then the canonical ensemble and isothermal isobaric ensemble (310 K, 1 standard atmospheric pressure) were then used to further balance the system for 100 ps. Finally, the molecular dynamics simulation was carried out at room temperature and atmospheric pressure for 100 ns.

In this study, the RMSD, root mean square fluctuation (RMSF), radius of gyration (Rg), number of hydrogen bonds between the protein and the compound in the complex (Hbonds), solvent-accessible surface area (SASA), Gibbs free energy landscape (FEL), energy of amino acid residues involved in binding (residue-energy) and average binding free energy (total-energy) were analyzed.

## 3. Results

### 3.1. Network pharmacology results

#### 3.1.1. Targets of the chemical ingredients of TXYF and UC-related targets

**Figure 2.**
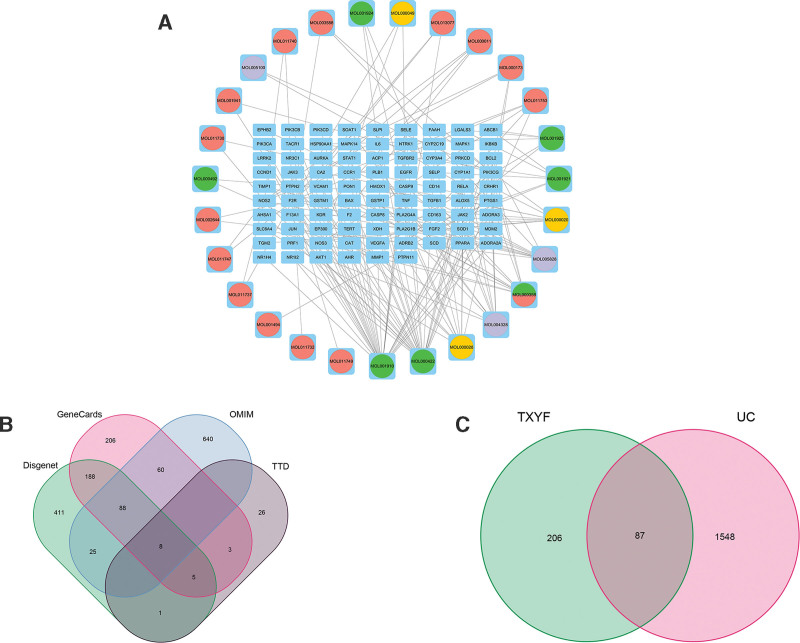
Correlation analysis of the targets of TXYF and UC. (A) Visualization network diagram of the intersection targets of TXYF and UC; (B) intersection plot displaying the UC targets retrieved from various databases; (C) Venn diagram of intersection targets between TXYF and UC. TXYF = Tongxie Yaofang, UC = ulcerative colitis.

A total of 43 chemical ingredients of TXYF and 206 related targets were selected. Moreover, 1548 UC-related targets were identified, as shown in Figure 2A. A comparison of the TXYF component targets and UC-related targets revealed 87 intersecting targets, as shown in Figure 2B. The drug and disease targets were imported into the Cytoscape 3.9.1 software for analysis, and a drug–disease–target network was constructed, as shown in Figure 2C. The analysis revealed that the components of Paeoniae Radix Alba had the greatest number of intersecting targets, with 11alpha,12alpha-epoxy-3beta-23-dihydroxy-30-norolean-20-en-28, 12beta-olide (MOL001910) having 29 targets, followed by kaempferol (MOL000422), which had 26 targets (Table 2).

**Table 2:** Drug components and corresponding targets.

Chinese name	Herbal name	MOL ID	Compound name	OB (%)	DL	Number of targets
Baishao	Paeoniae Radix Alba	MOL001910	11alpha,12alpha-epoxy-3beta-23-dihydroxy-30-norolean-20-en-28,12beta-olide	64.77	0.38	29
Baishao	Paeoniae Radix Alba	MOL000422	Kaempferol	41.88	0.24	26
Baizhu	Rhizoma Atractylodis Macrocephalae	MOL000028	α-Amyrin	39.51	0.76	12
Chenpi	Citri Reticulatae Pericarpium	MOL004328	Naringenin	59.29	0.21	12
Fangfeng and Baishao	Saposhnikoviae Radix and Paeoniae Radix Alba	MOL000358	Beta-sitosterol	36.91	0.75	11
Chenpi	Citri Reticulatae Pericarpium	MOL005828	Nobiletin	61.67	0.52	11
Baizhu	Rhizoma Atractylodis Macrocephalae	MOL000020	12-Senecioyl-2E,8E,10E-atractylentriol	62.4	0.22	9
Baishao	Paeoniae Radix Alba	MOL001921	Lactiflorin	49.12	0.8	6
Baishao	Paeoniae Radix Alba	MOL001925	Paeoniflorin_qt	68.18	0.4	5
Fangfeng	Saposhnikoviae Radix	MOL011753	5-O-Methylvisamminol	37.99	0.25	5
Fangfeng	Saposhnikoviae Radix	MOL000011	(2R,3R)-3-(4-hydroxy-3-methoxy-phenyl)-5-methoxy-2-methylol-2,3-dihydropyrano[5,6-h][1,4]benzodioxin-9-one	68.83	0.66	4
Fangfeng	Saposhnikoviae Radix	MOL000173	Wogonin	30.68	0.23	4
Baishao	Paeoniae Radix Alba	MOL001924	Paeoniflorin	53.87	0.79	3
Baizhu	Rhizoma Atractylodis Macrocephalae	MOL000049	3β-Acetoxyatractylone	54.07	0.22	3
Fangfeng	Saposhnikoviae Radix	MOL003588	Prangenidin	36.31	0.22	3
Fangfeng	Saposhnikoviae Radix	MOL013077	Decursin	39.27	0.38	3
Baishao	Paeoniae Radix Alba	MOL000492	(+)-Catechin	54.83	0.24	2
Chenpi	Citri Reticulatae Pericarpium	MOL005100	5,7-Dihydroxy-2-(3-hydroxy-4-methoxyphenyl)chroman-4-one	47.74	0.27	2
Fangfeng	Saposhnikoviae Radix	MOL011730	11-Hydroxy-sec-o-beta-d-glucosylhamaudol_qt	50.24	0.27	2
Fangfeng	Saposhnikoviae Radix	MOL011737	Divaricatacid	87	0.32	2
Fangfeng	Saposhnikoviae Radix	MOL011740	Divaricatol	31.65	0.38	2
Fangfeng	Saposhnikoviae Radix	MOL001941	Ammidin	34.55	0.22	2
Fangfeng	Saposhnikoviae Radix	MOL011747	Ledebouriellol	32.05	0.51	2
Fangfeng	Saposhnikoviae Radix	MOL002644	Phellopterin	40.19	0.28	2
Fangfeng	Saposhnikoviae Radix	MOL011732	Anomalin	59.65	0.66	1
Fangfeng	Saposhnikoviae Radix	MOL011749	Phelloptorin	43.39	0.28	1
Fangfeng	Saposhnikoviae Radix	MOL001494	Mandenol	42	0.19	1

*Note*: NR, not mentioned; n1, experimental group; n2, control group.

DL = drug likeness, OB = oral bioavailability.

#### 3.1.2. Key targets and protein interaction networks of TXYF in the treatment of UC

**Figure 3.**
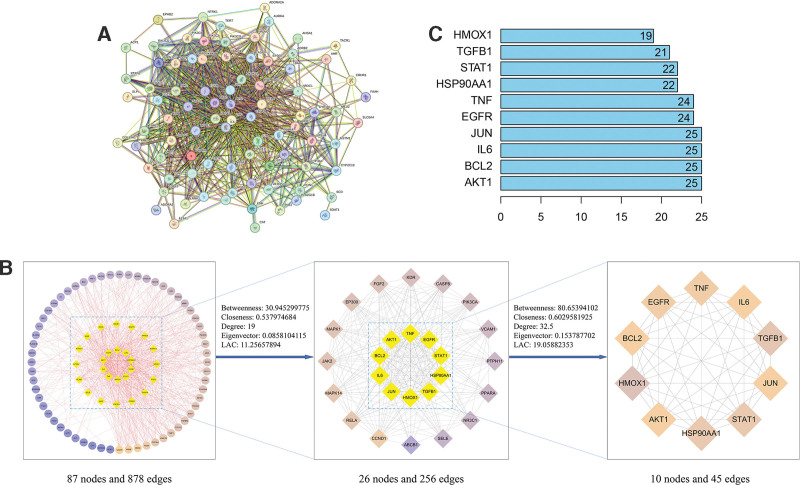
PPI network diagram and key target screening. (A) PPI network diagram; (B) flow chart of key target screening in the PPI network; (C) top 10 targets and corresponding degree values. PPI = protein–protein interaction.

We imported the 87 intersecting targets into the STRING database to construct a PPI network. Network analysis revealed 87 nodes and 878 protein interaction lines, with an average node degree of 20.2, as shown in Figure 3A. The PPI network data were imported into Cytoscape for drug–disease intersection target network visualization and topology analysis. Targets whose degree, closeness, betweenness, eigenvector, and local average connectivity values were greater than the median value were selected, and 10 key targets, namely, EGFR, HSP90AA1, JUN, BCL2, IL6, TNF, AKT1, HMOX1, TGFβ1, and STAT1, were ultimately selected. We speculated that TXYF may synergistically exert therapeutic effects through these 10 key targets. The results are displayed in Table 3, Figure 3B and C.

**Table 3:** Topological score of key targets.

Target	Betweenness	Closeness	Degree	Eigenvector	LAC
EGFR	483.52785	0.7142857	53	0.2070101	20.603774
HSP90AA1	241.42283	0.6640625	45	0.1875859	20.355556
JUN	140.88085	0.68	47	0.2037967	22.808511
BCL2	192.28034	0.7024793	50	0.2108101	22.76
IL6	849.30507	0.8252427	67	0.234614	20.626866
TNF	836.74491	0.8252427	67	0.2336627	20.38806
AKT1	580.31173	0.7798165	61	0.2277661	21.377049
HMOX1	80.704455	0.615942	34	0.1548838	19.117647
TGFβ1	135.72758	0.648855	43	0.1832346	20.511628
STAT1	89.600205	0.625	37	0.1690203	20.594595

LAC = local average connectivity.

#### 3.1.3. GO and KEGG enrichment analyses

(1) Gene ontology (GO) enrichment analysis.

**Figure 4.**
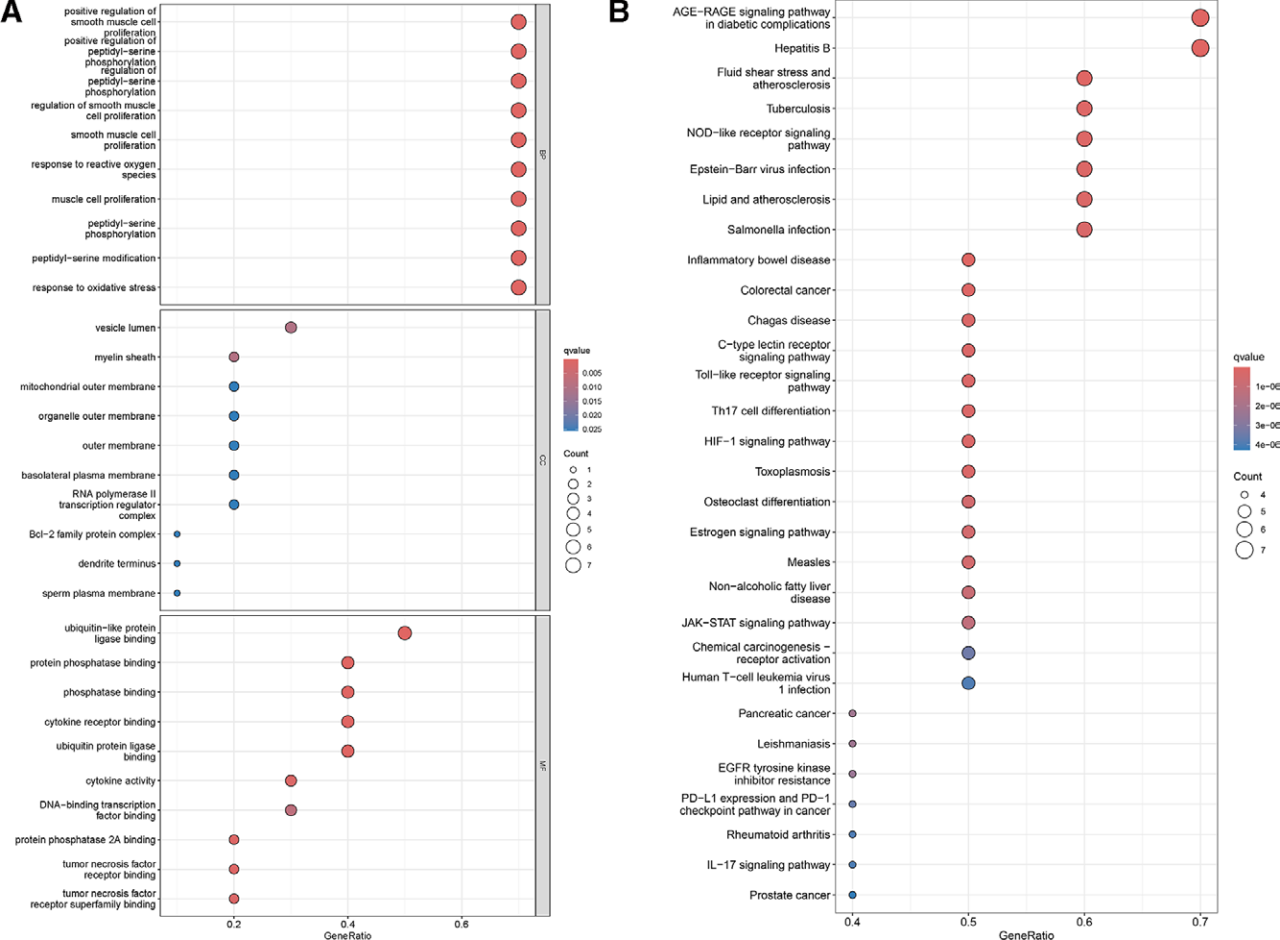
GO and KEGG enrichment analysis results. (A) GO enrichment analysis; (B) KEGG enrichment analysis. GO = gene ontology, KEGG = Kyoto Encyclopedia of Genes and Genomes.

After the potential targets were analyzed, 1586 GO enrichment terms were obtained. A total of 1491 biological process (BP), 33 cellular component, and 62 molecular function terms were identified. The potential BP affected by TXYF in the treatment of UC included the regulation of smooth muscle cell proliferation, protein kinase regulation, metabolic process regulation, epithelial cell proliferation, and the oxidative stress response (Fig. 4A).

(2) KEGG enrichment analysis and visualization of the drug pathway target network.

We identified a total of 122 KEGG pathways associated with potential targets for the treatment of UC with TXYF. Multiple signaling pathways, including inflammatory bowel disease, NOD-like receptor, colorectal cancer, Toll-like receptor, Th17 cell differentiation, and the HIF-1 and JAK/STAT pathways, are involved. The results are displayed in Figure 4B. Thus, TXYF exerts therapeutic effects on UC through multiple pathways.

**Figure 5.**
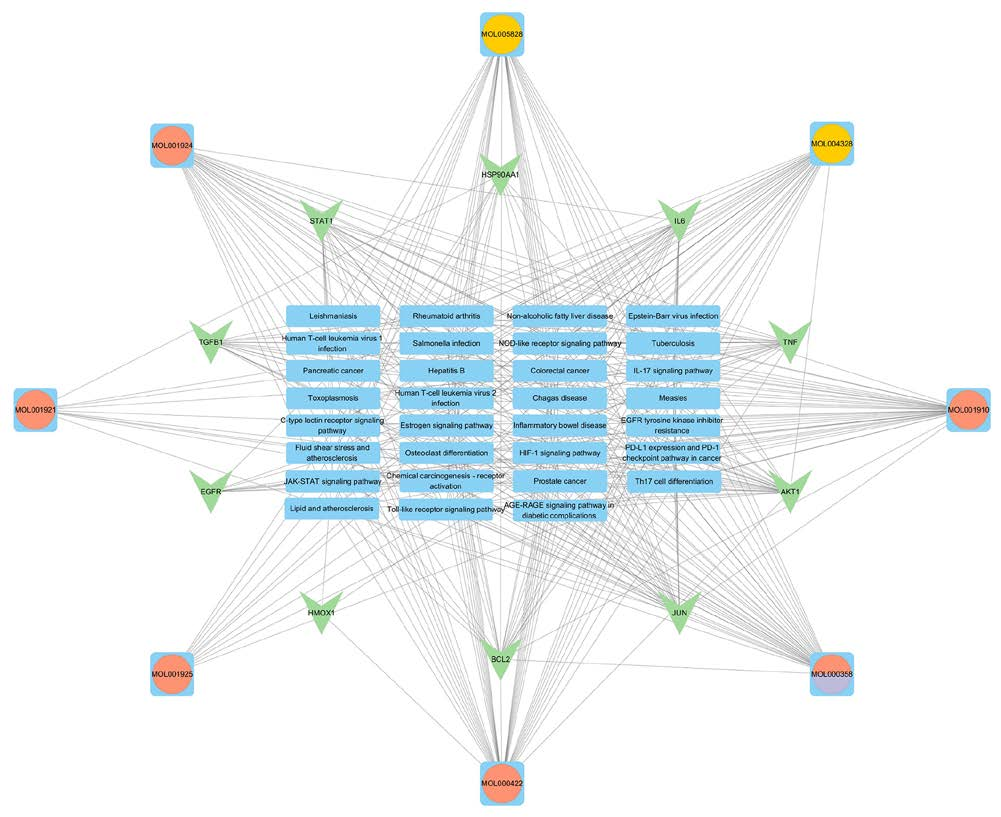
Chemical ingredient-pathway-key target network.

By importing the data on the drug components, related signaling pathways, and core targets into the Cytoscape software for drug–pathway–target network analysis and visualization, we found that the TXYF components Paeoniae Radix Alba, Saposhnikoviae Radix, and Citri Reticulatae Pericarpium had interactive regulatory effects on multiple signaling pathways and key targets, as shown in Figure 5.

### 3.2. Molecular docking results

**Figure 6.**
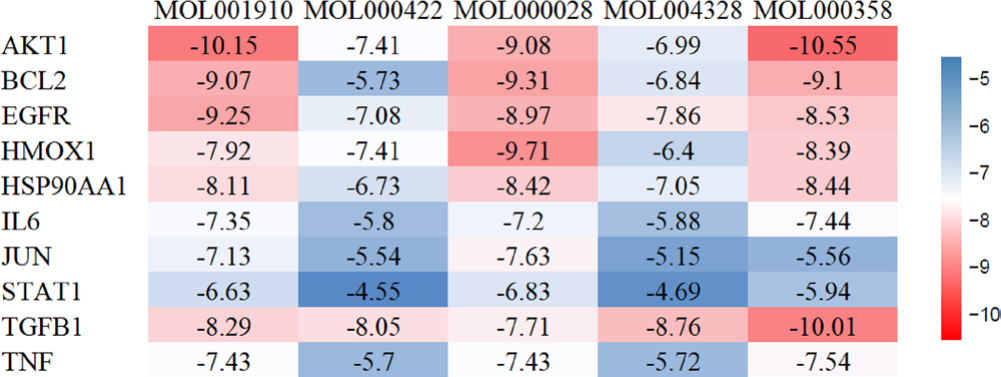
Heatmap of the binding energies.

**Figure 7.**
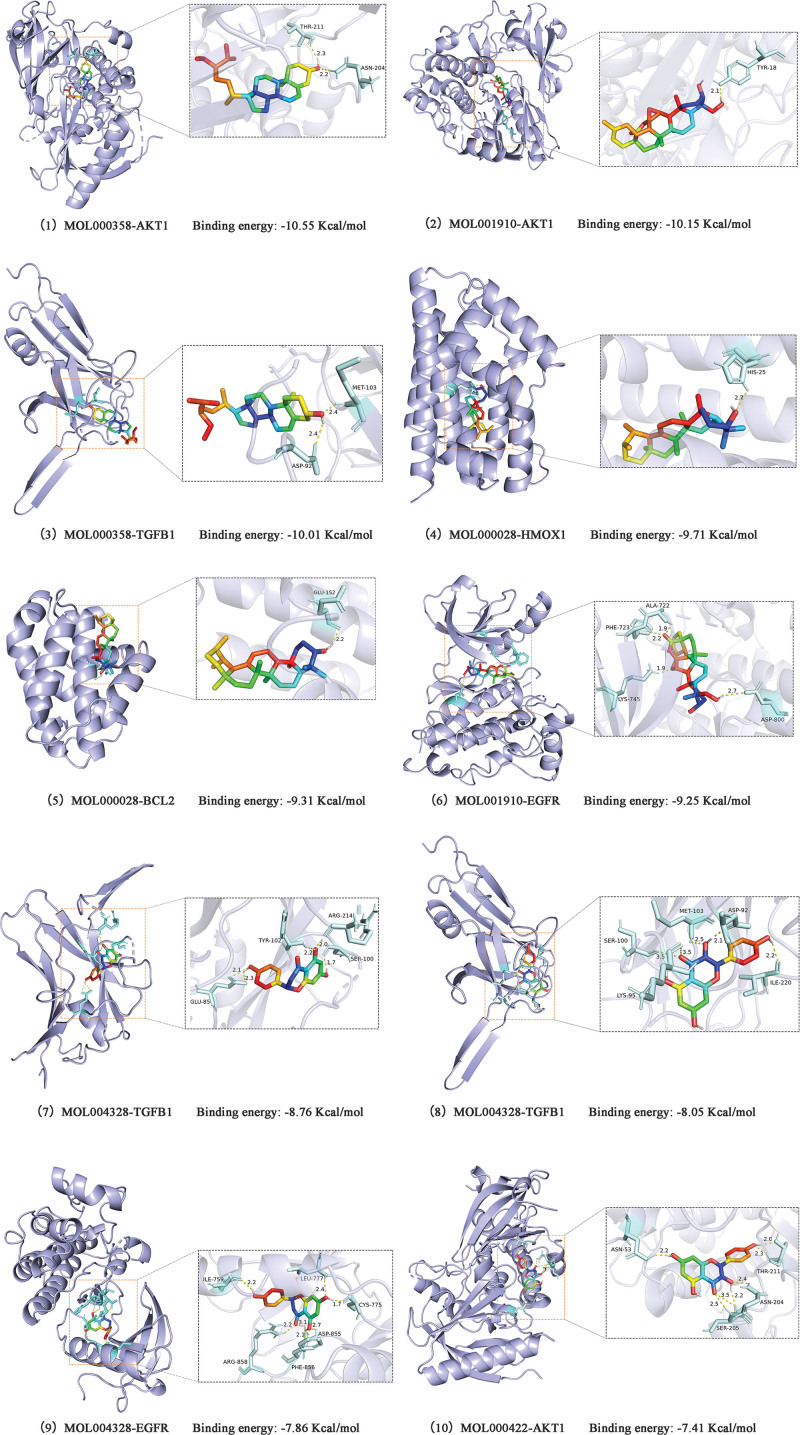
Molecular docking results.

Based on the analysis of the drug–disease network and PPI networks, molecular docking analysis was performed on the top 5 chemical ingredients and the top 10 core targets of TXYF. The docking results are presented as heatmaps in Figure 6. The docking results for 5 chemical ingredients with 2 targets that had the strongest binding energies were visualized, as shown in Figure 7. The lower the docking fraction between the chemical ingredient and protein was, the stronger the binding force was. A docking score of <1.2 kcal/mol indicated a strong docking effect.^[13]^ The results revealed that the binding energies of AKT1 (PDB: 7NH5), BCL2 (PDB: 6O0P), EGFR (PDB: 8A2D), HMOX1 (PDB: 6EHA), HSP90AA1 (PDB: 7UR3), and TGFβ1 (PDB: 6P7J) to the chemical ingredients of the drugs were relatively stable, suggesting that these proteins may be potential targets for the treatment of UC with TXYF. Among them, AKT1 had the highest binding energy (‐10.55 kcal/mol).

### 3.3. Molecular dynamics simulation results

#### 3.3.1. Methodological verification results

**Figure 8.**
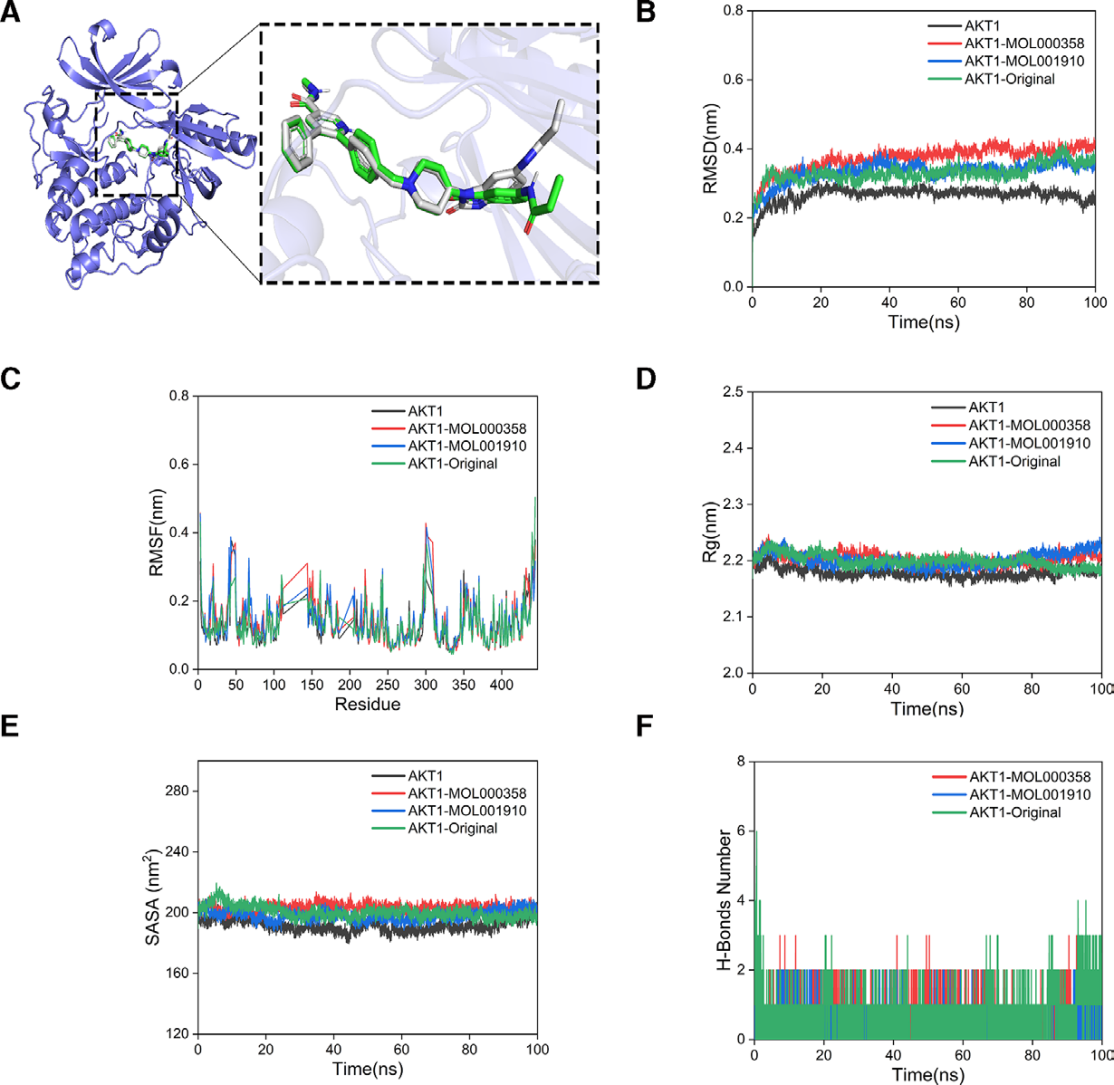
Analysis results of molecular dynamics simulations. (A) Methodological verification; (B) RMSD analysis; (C) RMSF analysis; (D) Rg analysis; (E) SASA analysis; (F) hydrogen bond analysis. Rg = radius of gyration, RMSD = root mean square deviation, RMSF = root mean square fluctuation, SASA = solvent-accessible surface area.

The 2 molecular complexes with the strongest binding energies were those of the AKT1 protein with MOL000358 (‐10.55 kcal/mol) and MOL001910 (‐10.15 kcal/mol). The eutectic ligand bound to the AKT1 protein was used to bind to the AKT1 protein again. As shown in Figure 8A, the whole structure of the eutectic ligand before and after docking mostly overlapped, and the RMSD value was 0.31, which indicated that the method was verified.

#### 3.3.2. Molecular dynamics simulation grouping

Because semiflexible molecular docking cannot currently consider the flexibility of the protein structure at present, to further prove the degree and stability of the interaction between the compound and protein, this study carried out molecular dynamics simulations of the complexes of the AKT1 protein with MOL000358 and MOL001910 for 100 ns and compared them with the uncomplexed AKT1 protein as the control group and AKT1 in complex with the eutectic ligand as the positive control group to study the binding stabilities of the AKT1 protein with MOL000358 and MOL001910.

#### 3.3.3. RMSD and RMSF analyses

(1) RMSD analysis.

The RSMD is an index used to assess the stability of a protein–ligand complex. The smaller the RMSD is, the smaller the overall structural change in the complex is, and the more stable the complex is. In Figure 8B, the RMSD curve of the AKT1 blank group has the smallest fluctuation range, and the RMSD curve of the MOL001910 group almost overlaps with that of the positive control group of the eutectic ligand, while the RMSD curve of the MOL000358 group fluctuates slightly. These results demonstrate that the complex formed by MOL001910 and the AKT1 protein is very stable compared with that of the blank group and the positive control group, and the stability is close to that of the eutectic ligand complex. The stability of the complex formed by MOL000358 and AKT1 was slightly lower, but the RMSD curve did not greatly fluctuate, and the fluctuation range was within 1 nm, indicating that MOL000358 and AKT1 also formed a very stable complex.

(2) RMSF analysis.

The RMSF represents the degree of fluctuation of amino acid residues in protein during kinetic simulation. A high RMSF value represents a large fluctuation of the amino acid residues, whereas a low RMSF value represents a small fluctuation. In Figure 8C, the 4 groups of RMSF curves almost overlap, the fluctuations are all within the range of 1 nm, and there is no large fluctuation. The above results showed that the addition of MOL000358, MOL001910 and the eutectic ligand had little effect on the stability of amino acid residues in the AKT1 protein, and the formed complexes were all very stable.

#### 3.3.4. Rg analysis and SASA analyses

(1) Rg analysis.

The Rg is used to characterize the compactness and stability of the structure in a dynamic simulation. A larger Rg indicates that the structure has substantially expanded; conversely, a smaller Rg indicates that the structure remains tight and stable. As shown in Figure 8D, the fluctuation ranges of the 4 groups of Rg curves were all approximately 2.2 nm, the entire process was stable, without large fluctuations, and the 4 groups of curves almost overlapped. These findings showed that the complexes formed by the AKT1 protein with MOL000358, MOL001910, and the eutectic ligand were all tight and stable, and that the addition of MOL000358, MOL001910 or the eutectic ligand did not greatly change the overall structure of the protein.

(2) SASA analysis.

The SASA can be used to evaluate protein structure folding and stability. Stable SASA curves indicate that the proteins have stable structures. Figure 8E shows that the 4 groups of SASA curves were stable throughout the whole process, and the fluctuation range was approximately 200 nm^2^, with no significant fluctuations. Compared with that of the blank group, the accessible surface area of the protein solvent slightly increased with the addition of MOL000358, MOL001910 or the eutectic ligand, but the stability was not much different. Compared with that of the positive control group, the SASA curve of the MOL001910 group had slightly lower fluctuations and a better stability. The above results revealed that the AKT1 protein formed highly stable complexes with MOL000358, MOL001910 and the eutectic ligand, among which the complex of the AKT1 protein and MOL001910 had the greatest stability.

#### 3.3.5. Hydrogen bond analysis

To study the hydrogen bonding properties of the complex binding sites, the number of hydrogen bonds between a ligand and the protein was calculated. Figure 8F shows that the number of hydrogen bonds formed between the eutectic ligand and the AKT1 protein was stable at 1 to 2, and the number of hydrogen bonds formed between MOL000358 or MOL001910 and AKT1 fluctuated at approximately 1 to 2, indicating that similar to the positive control group, MOL000358 or MOL001910 and AKT1 also formed relatively stable hydrogen bond interactions, and the complexes were very stable.

#### 3.3.6. FEL analysis

**Figure 9.**
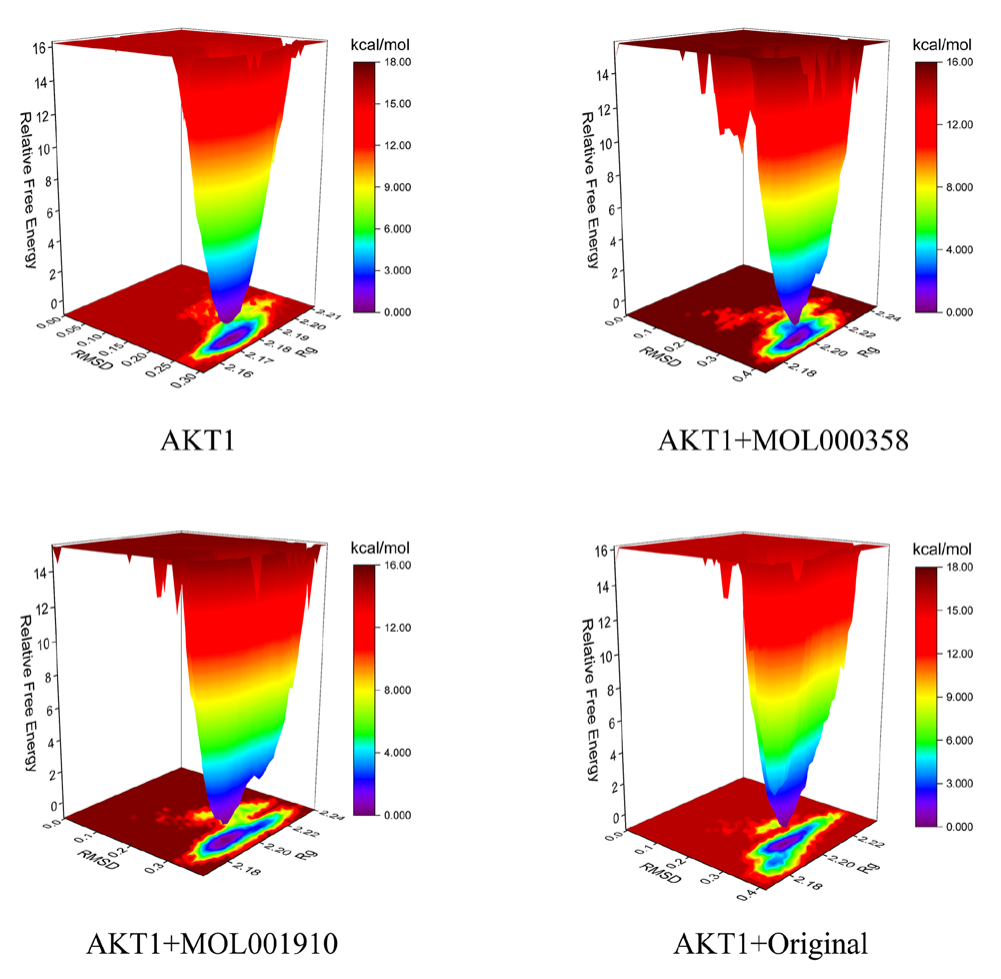
FEL map of the complex. FEL = Gibbs free energy landscape.

The FEL is used to estimate the conformation with the lowest energy during the whole process of complex structural dynamics simulation. A weak and unstable interaction between the protein and a ligand results in many minimum energy clusters with rough surfaces in the FEL diagram. In contrast, the energy clusters formed by strong and stable interactions are close to being single and smooth in the FEL diagram. In Figure 9, the dark purple/blue dots reflect the minimum energy, which indicates the most stable structure, and the red/yellow dots represent unstable structures. Figure 9 shows that the minimum energy cluster formed in the free energy distribution diagram of the AKT1 blank group has the best single and smooth degree, indicating the highest stability, whereas the other 3 groups of energy clusters have generated many smaller clusters; however, the clusters are concentrated, and the surface is not rough, which indicates that the complexes formed between the AKT1 protein and MOL000358, MOL001910 and the eutectic ligand have good stability.

#### 3.3.7. Total energy

**Figure 10.**
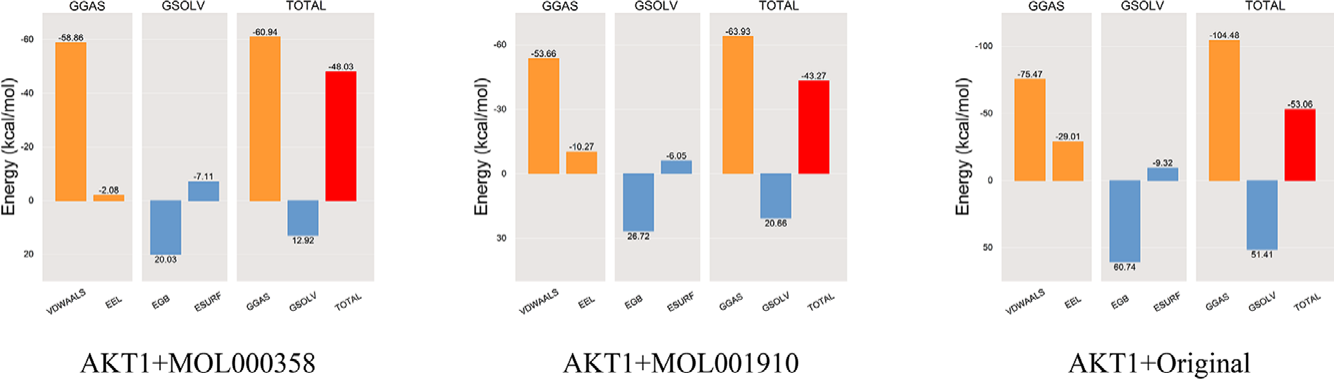
Total-energy of the complex. VDWAALS, EEL, EGB, ESURF, GGAS, GSOLV, and TOTAL represent the van der Waals force, electrostatic energy, polar solvation energy, nonpolar solvation energy, molecular mechanics term, solvation energy term and average binding free energy, respectively.

After the complex system stabilized, the total energy between the small-molecule ligand and the protein receptor was calculated to reflect the binding affinity and degree between the protein and the ligand. The system with low total energy is more stable. We calculated the binding free energies of the AKT1 protein complexes with MOL000358, MOL001910, and the eutectic ligand. Figure 10 shows that the total energies of the AKT1 protein complexes with MOL000358, MOL001910, and the eutectic ligand were ‐48.03, ‐43.27 and ‐53.06 kcal/mol, respectively, indicating that the degrees of binding of the AKT1 protein with MOL00358 and MOL001910 were slightly weaker than those in the positive control group but still strong.

#### 3.3.8. Energy of amino acid residues involved in binding

**Figure 11.**
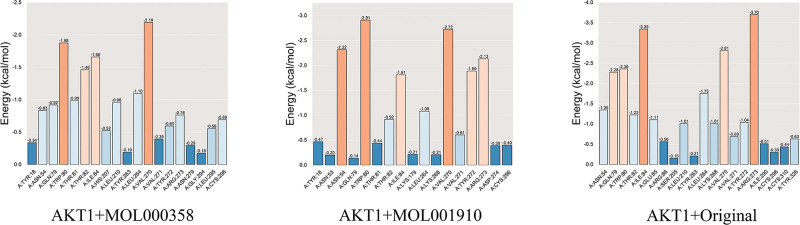
Residue energy of amino acids involved in binding.

By calculating the contributions of the residue energy of the amino acids involved in binding, we determined the specific amino acids that bind to small-molecule ligands and the corresponding binding energies and further analyzed the contributions of the amino acid residues to the binding energies. Figure 11 shows that the amino acid residues of the AKT1 protein bound to MOL000358, MOL001910, and the eutectic ligand almost overlap, and the ligands interact mainly with amino acids such as TRP-80, ILE-84, VAL-270, and ARG-273. The results revealed that amino acids such as TRP-80, ILE-84, VAL-270, and ARG-273 are the common binding sites and are likely to be the key sites for inhibiting AKT1.

## 4. Discussion

UC is a chronic nonspecific colitis disease, and its clinical symptoms are prone to recurring. In recent years, the incidence of UC has significantly increased.^[2]^ The etiology of UC has not yet been determined, and research has suggested that this disease may be related to factors such as genetics, the living environment, autoimmune factors, infections, and psychological factors; this leads to the inability of conventional WM to achieve good therapeutic effects. In addition, steroid drugs can cause adverse reactions, drug resistance, and drawbacks, such as high costs and easy disease recurrence after medication, making the treatment of UC challenging. Therefore, research has focused mostly on finding treatment methods to maintain UC remission, reduce adverse reactions during UC treatment, and reduce recurrence rates.^[3]^

TXYF is a classical TCM formula for treating diarrhea and is commonly used to treat digestive system diseases with symptoms of diarrhea, including UC and IBS.^[8]^ However, TXYF is currently used primarily for the treatment of diarrhea-induced IBS; its efficacy in treating UC is somewhat controversial, and the molecular mechanism of TXYF in treating UC is currently unclear. Therefore, this study integrated a meta-analysis with network pharmacology and molecular dynamics simulations to explore the efficacy, safety, potential molecular mechanisms, potential chemical ingredients, key targets, and binding stability between key targets and TXYF ligands in treating UC.

### 4.1. TXYF can act on UC through multiple targets and pathways

TXYF consists of 4 medicines, namely, Saposhnikoviae Radix, Paeoniae Radix Alba, Rhizoma Atractylodis Macrocephalae, and Citri Reticulatae Pericarpium. Research has shown that Saposhnikoviae Radix extract regulates immune function, protects the intestinal cell barrier, and prevents diarrhea. The ingredients contained in Saposhnikoviae Radix have analgesic, antioxidant, and anti-inflammatory effects, as well as inhibitory effects on the allergic contraction of intestinal smooth muscles. This treatment can also decrease the number of inflammatory cells in UC colon tissue and alleviate inflammatory reactions and intestinal mucosal damage.^[14]^ Both Rhizoma Atractylodis Macrocephalae and Paeoniae Radix Alba can alleviate intestinal mucosal inflammation and regulate immunity by regulating Th17 cell differentiation and maintaining Th17 cell/Treg cell immune homeostasis.^[15,16]^ These 2 types of drugs also regulate the abundance of the intestinal microbiota and improve gastrointestinal function.^[17]^ Paeoniae Radix Alba extract can also protect and repair the intestinal barrier by affecting Toll-like receptor 4 (TLR4)-dependent oxidative stress and inflammatory signaling.^[18,19]^ Research has shown that Citri Reticulatae Pericarpium has anticancer, anti-inflammatory, and antioxidant effects.^[20,21]^ A recent animal experimental study revealed that TXYF can reduce the expression of the inflammatory factors IL-17 and TNF-α in a UC rat model by regulating the MAPK/AKT signaling pathway, which shows that TXYF can alleviate the inflammatory reaction in UC.^[22]^ These studies provide pharmacological evidence for the treatment of UC with TXYF, but the specific molecular mechanism of action of TXYF is unclear.

To further explore the mechanism of action of TXYF in treating UC, we used network pharmacology to predict the chemical ingredients of TXYF and the key targets, pathways, and other molecular mechanisms involved in UC treatment. This study identified a total of 43 chemical ingredients and 206 targets of TXYF, as well as 1548 disease targets related to UC. We selected 87 cross-targets from 206 targets of the chemical ingredients of TXYF and 1548 UC-related targets. According to the drug–disease–target network, the 5 most common chemical ingredients of TXYF in the treatment of UC include 11alpha,12alpha-epoxy-3beta-23-dihydroxy-30-norolean-20-en-28,12beta-olide (MOL001910) and kaempferol (MOL000422), which are components of Paeoniae Radix Alba; α-amyrin (MOL000028), which is a component of Rhizoma Atractylodis Macrocephalae; naringenin (MOL004328), which is a component of Citri Reticulatae Pericarpium; and β-sitosterol (MOL000358), which is a chemical ingredient shared by Saposhnikoviae Radix and Paeoniae Radix Alba.

Due to the complexity of TCM ingredients, the specific mechanism of action of TXYF in treating UC has not yet been determined. Therefore, we conducted KEGG and GO enrichment analyses of the cross-target effects of UC and TXYF to further explore the underlying therapeutic mechanism involved. We found that the mechanism by which TXYF treats UC is related to various BPs, such as metabolic process regulation, epithelial cell proliferation, and the oxidative stress response, as well as multiple signaling pathways, such as the AGE/RAGE, TOLL, Th17, and JAK/STAT pathways.

Research has shown that RAGE levels increase in IBD patients, and that advanced glycation end products (AGEs) and RAGE can induce the production of reactive oxygen species, which can regulate the oxidative stress response and the NF-κB signaling pathway and promote TNF-α expression, ultimately leading to an inflammatory response and tissue damage.^[23–25]^ RAGE blockade or RAGE gene knockout can not only prevent DSS-induced diarrhea but also alleviate colitis invasion in experimental animals.^[26–28]^ Animal experiments have shown that kaempferol can regulate the AGE–RAGE signaling pathway in DSS-induced colitis mice and that reducing the activation of the NF-κB signaling pathway reduces the inflammatory response.^[29,30]^

Toll-like receptors are receptors that mediate the innate immune response. The expression of TLR4 is positively correlated with disease severity in UC patients.^[31–33]^ Kaempferol can alleviate UC-related intestinal inflammation by regulating the IL-17 and TLR4 signaling pathways.^[34,35]^ Kaempferol downregulates TNF-α and IL-1β in both serum and colon tissue by reducing TLR4 levels. In addition to regulating the expression of the inflammatory marker IL-6, kaempferol can also regulate plasma NO levels and colon MPO activity in DSS-treated mice through the TLR4/STAT signaling pathway, thereby reducing UC-related oxidative stress and the inflammatory response and alleviating intestinal mucosal damage.^[36,37]^ Moreover, kaempferol can also improve colitis in mice by regulating metabolic processes such as arginine, galactose, and proline metabolism.^[33,34]^ An animal experimental study suggested that kaempferol and beta-sitosterol could alleviate intestinal inflammation and protect the intestinal barrier by regulating the Toll-like receptor pathway.^[33]^ β-Sitosterol not only downregulates TLR4 levels but also alleviates oxidative stress and inflammatory cascade reactions, thereby restoring intestinal mucosal barrier integrity.^[38]^

According to previous reports, IBD patients may have elevated levels of STAT3 or STAT3 phosphorylation.^[39,40]^ JAK signal transduction can affect the expression of various proinflammatory cytokines and is one of the driving factors of UC. Related studies have shown that the expression of JAK2, JAK1, and TYK2 is significantly increased in the intestinal epithelial cells of UC patients.^[41]^ STAT5 can promote Treg cell proliferation, regulate immune balance, and alleviate the intestinal inflammatory response in UC patients.^[29,42]^ IL-6 can activate STAT3 and regulate the immune balance of Th17 cells/Treg cells and CD4 + T cells. The level of IL-17 secreted by Th17 cells is closely related to the severity of UC and can synergistically activate NF-κB via the AGE pathway.^[43,44]^ β-Sitosterol has anti-inflammatory activity, can regulate the Th17 cell/Treg cell immune balance, and can reduce the levels of the inflammatory factors IL-6 and TNF-α to alleviate UC-related intestinal inflammation.^[45]^ Multiple studies have shown that beta-sitosterol has antipathogenic effects, reduces leukocyte infiltration and the expression of apoptotic factors, and decreases IL-1β, IL-6 and TNF-α levels.^[46]^ The expression of inflammatory factors can also protect intestinal barrier function and reduce intestinal mucosal damage.^[47,48]^ These studies suggest that the drug components contained in TXYF can effectively act against UC.

GO and KEGG analyses indicated that TXYF could exert therapeutic effects on UC through multiple biological functions and signaling pathways, and the PPI network and molecular docking results further clarified the targets of TXYF in the treatment of UC. The results showed that AKT1, BCL2, EGFR, HMOX1, HSP90AA1, and TGFβ1 were potential targets through which TXYF could treat UC. AKT1 and TGFβ1 exhibit high binding energies with beta-sitosterol. AKT1 can also participate in regulating the AGE/RAGE, TOLL, and JAK/STAT signaling pathways, whereas TGFβ1 can participate in regulating the Th17 signaling pathway. Based on the above results, we speculated that TXYF may exert therapeutic effects on UC mainly through the AGE/RAGE, TOLL, JAK/STAT, and Th17 signaling pathways. In summary, TXYF is a safe and effective prescription for treating UC and can exert therapeutic effects through multiple pathways and targets. The therapeutic mechanism may involve inflammation, oxidative stress, cell proliferation, and immune cell regulation. These findings can provide a reference for the selection of drugs for the treatment of UC; however, this study did not address the issue of drug dosage, which is a limitation. In the future, further clinical research and basic experiments are needed to verify the relevant mechanisms of TXYF in treating UC.

### 4.2. Chemical ingredients of TXYF can be stably combined with the key target AKT1 to treat UC

The molecular docking results revealed that the complexes of the AKT1 protein with MOL000358 (‐10.55 kcal/mol) and MOL001910 (‐10.15 kcal/mol) had the strongest binding energies. To provide a reference for the application of TXYF, the treatment of UC and future basic experimental research are needed. Based on the results of molecular docking, we selected the key Akt1 protein and the TXYF chemical components MOL000358 and MOL001910 for molecular dynamics simulations to further verify the binding ability and stability of drugs to disease targets and analyze the molecular mechanism of TXYF in treating UC.

For the molecular dynamics simulations, the uncomplexed AKT1 protein was used as the control group, and the complex of AKT1 with the eutectic ligand was compared with the positive control group to study the binding stabilities of the AKT1 protein complexes with MOL000358 and MOL001910. Through molecular dynamics simulation analysis of each group, the RMSD, RMSF, Rg, SASA, number of hydrogen bonds between the protein and the compound, FEL and average binding free energy results revealed that the complexes formed by the chemical TXYF ingredients MOL001910 and MOL00035 and the AKT1 protein had good stability and high binding strength, could form relatively stable hydrogen bond interactions, and did not affect amino acid residues or the whole AKT1 protein. Analysis of the energy of the amino acid residues involved in binding suggested that the common binding sites of MOL000358 and MOL001910 to the AKT1 protein were amino acids such as TRP-80, ILE-84, VAL-270, and ARG-273, which are likely the key sites for inhibiting AKT1. The above results indicate that TXYF may play a therapeutic role in treating UC mainly through 2 chemical ingredients, β-sitosterol (MOL000358) and 11alpha,12alpha-epoxy-3beta-23-dihydroxy-30-norolean-20-en-28,12beta-olide (MOL001910), and that AKT1 may be the most critical target of TXYF in treating UC.

In this study, the potential therapeutic targets, chemical components and pathways of TXYF in the treatment of UC were identified; however, there are several limitations exist. First, the pharmacokinetics of the chemical components of TXYF, obtained through database information and computer predictions, remain to be tested in the human body. Moreover, the specific mechanism of action of TXYF in treating UC still requires further animal or cell experiments for verification.

## 5. Conclusion

In this study, we analyzed the mechanism of action of TXYF in treating UC. These results indicate that the mechanism of action of TXYF in treating UC may be related to the regulation of inflammation, oxidative stress, cell proliferation, and immune responses. The AGE/RAGE, TOLL, JAK/STAT, and Th17 signaling pathways may be important signaling pathways for the treatment of UC with TXYF. The targets of TXYF for treating UC may include AKT1, BCL2, EGFR, HMOX1, HSP90AA1, and TGFβ1. Among them, AKT1 has the highest binding energy (‐10.55 kcal/mol). Molecular dynamics simulations revealed that the key chemical components of TXYF in the treatment of UC may be β-sitosterol (MOL000358) and 11alpha,12alpha-epoxy-3beta-23-dihydroxy-30-norolean-20-en-28,12 beta-olide (MOL001910). The complexes formed by the AKT1 protein and the effective TXYF components MOL001910 and MOL00035 have good stability and high binding strength. Therefore, AKT1 may be the most critical target of TXYF in the treatment of UC.

## Author contributions

**Conceptualization:** Lili Tang.

**Data curation:** Lili Tang, Hongwu Tao.

**Formal analysis:** Lili Tang, Hongwu Tao.

**Investigation:** Wenzhe Feng.

**Methodology:** Yuedong Liu.

**Project administration:** Yuedong Liu.

**Supervision:** Yuedong Liu.

**Writing – original draft:** Lili Tang.

**Writing – review & editing:** Yuedong Liu, Hongwu Tao, Wenzhe Feng, Cong Ren.

## References

[1] Mo X Tang K Deng L. Prevention of ulcerative colitis by Huangqin decoction: reducing the intestinal epithelial cell apoptosis rate through the IFN-γ/JAK/ETS signalling pathway. Pharm Biol. 2022;60:1116–25. 35654745 10.1080/13880209.2022.2070220 PMC9176677

[2] Gu Z Chen X Zhu D Wu S Yu C. Histone deacetylase 1 and 3 inhibitors alleviate colon inflammation by inhibiting Th17 cell differentiation. J Clin Lab Anal. 2022;36:e24699. 36106389 10.1002/jcla.24699 PMC9550981

[3] Krugliak Cleveland N Torres J Rubin DT. What does disease progression look like in ulcerative colitis, and how might it be prevented? Gastroenterology. 2022;162:1396–408. 35101421 10.1053/j.gastro.2022.01.023

[4] Liu Y Li BG Su YH. Potential activity of traditional Chinese medicine against ulcerative colitis: a review. J Ethnopharmacol. 2022;289:115084. 35134488 10.1016/j.jep.2022.115084

[5] He BY Wang PR Yang WZ. Inflammatory factor level, intestinal flora distribution and related factors in patients with ulcerative colitis. S China J Prev Med. 2022;48:178–81.

[6] Aslam N Lo SW Sikafi R. A review of the therapeutic management of ulcerative colitis. Therap Adv Gastroenterol. 2022;15:17562848221138160. 10.1177/17562848221138160 PMC9720837 36478780

[7] Wang M Fu RJ Xu DQ. Traditional Chinese medicine: a promising strategy to regulate the imbalance of bacterial flora, impaired intestinal barrier and immune function attributed to ulcerative colitis through intestinal microecology. J Ethnopharmacol. 2024;318:116879. 37419224 10.1016/j.jep.2023.116879

[8] Zhang S Wu ZJ Fan YB. Clinical effect of Tongxie Yaofang granule on diarrhea irritable bowel syndrome with liver depression and spleen deficiency. Chin Tradit Pat Med. 2023;45:3509–12.

[9] Zhou HW Wang JJ Chi L. Clinical research progress of spleen-invigorating prescriptions in treating ulcerative colitis. Chin J Lib Inf Sci Tradit Chin Med. 2022;46:71–6.

[10] Zhao L Zhang H Li N. Network pharmacology, a promising approach to reveal the pharmacology mechanism of Chinese medicine formula. J Ethnopharmacol. 2023;309:116306. 36858276 10.1016/j.jep.2023.116306

[11] Sun TL Quan WJ Peng S. Network pharmacology-based strategy combined with molecular docking and in vitro validation study to explore the underlying mechanism of Huo Luo Xiao Ling Dan in treating atherosclerosis. Drug Des Devel Ther. 2022;Volume 16:1621–45. 10.2147/DDDT.S357483 PMC9166517 35669282

[12] Ye JH Li L Hu ZX. Exploring the molecular mechanism of action of Yinchen Wuling powder for the treatment of hyperlipidemia, using network pharmacology, molecular docking, and molecular dynamics simulation. Biomed Res Int. 2021;2021:1–14. 34746316 10.1155/2021/9965906 PMC8568510

[13] Shahnawaz Khan M Tabrez S Rehman MT Alokail MS. Al (III) metal augment thermal aggregation and fibrillation in protein: role of metal toxicity in neurological diseases. Saudi J Biol Sci. 2020;27:2221–6. 32874119 10.1016/j.sjbs.2020.05.013 PMC7451595

[14] Jia Q Sun W Zhang L. Screening the anti-allergic components in Saposhnikoviae Radix using high-expression Mas-related G protein-coupled receptor X2 cell membrane chromatography online coupled with liquid chromatography and mass spectrometry. J Sep Sci. 2019;42:2351–9. 31050150 10.1002/jssc.201900114

[15] Wang FY Su M Zheng YQ. Herbal prescription Chang’an II repairs intestinal mucosal barrier in rats with post-inflammation irritable bowel syndrome. Acta Pharmacol Sin. 2015;36:708–15. 25960135 10.1038/aps.2014.170 PMC4594184

[16] Chen K Lou Y Zhu Y. Tong Xie Yao Fang: a classic Chinese medicine prescription with potential for the treatment of ulcerative colitis. Evid Based Complement Alternat Med. 2021;2021:5548764. 34211567 10.1155/2021/5548764 PMC8208878

[17] Cheng H Zhang D Wu J. Atractylodes macrocephala Koidz. volatile oil relieves acute ulcerative colitis via regulating gut microbiota and gut microbiota metabolism. Front Immunol. 2023;14:1127785. 37205093 10.3389/fimmu.2023.1127785 PMC10187138

[18] Zheng K Jia J Yan S Shen H Zhu P Yu J. Paeoniflorin ameliorates ulcerative colitis by modulating the dendritic cell-mediated T(H)17/T(reg) balance. Inflammopharmacology. 2020;28:1705–16. 32472435 10.1007/s10787-020-00722-6 PMC7572351

[19] Liu B Piao X Niu W. Kuijieyuan decoction improved intestinal barrier injury of ulcerative colitis by affecting TLR4-dependent PI3K/AKT/NF-κB oxidative and inflammatory signaling and gut microbiota. Front Pharmacol. 2020;11:1036. 32848725 10.3389/fphar.2020.01036 PMC7403404

[20] Chang Y Zhai L Peng J Wu H Bian Z Xiao H. Phytochemicals as regulators of Th17/Treg balance in inflammatory bowel diseases. Biomed Pharmacother. 2021;141:111931. 34328111 10.1016/j.biopha.2021.111931

[21] Yang M Zhang Q Taha R. Polysaccharide from atractylodes macrocephala Koidz. ameliorates DSS-induced colitis in mice by regulating the Th17/Treg cell balance. Front Immunol. 2022;13:1021695. 36341374 10.3389/fimmu.2022.1021695 PMC9630481

[22] Liu X Ye M He Y Lai Q Liu B Zhang L. Investigation of Tongxie-Yaofang formula in treating ulcerative colitis based on network pharmacology via regulating MAPK/AKT signaling pathway. Aging (Milano). 2024;16:1911–24. 10.18632/aging.205467 PMC10866423 38271090

[23] Fukami K Yamagishi S Okuda S. Role of AGEs-RAGE system in cardiovascular disease. Curr Pharm Des. 2014;20:2395–402. 23844818 10.2174/13816128113199990475

[24] Moura FA Goulart MOF Campos SBG da Paz Martins AS. The close interplay of nitro-oxidative stress, advanced glycation end products and inflammation in inflammatory bowel diseases. Curr Med Chem. 2020;27:2059–76. 30182837 10.2174/0929867325666180904115633

[25] Niu K Li Q Liu Y. Molecular targets and mechanisms of scutellariae radix-coptidis rhizoma drug pair for the treatment of ulcerative colitis based on network pharmacology and molecular docking. Evid Based Complement Alternat Med. 2021;2021:9929093. 34149863 10.1155/2021/9929093 PMC8195671

[26] Chang JS Wendt T Qu W. Oxygen deprivation triggers upregulation of early growth response-1 by the receptor for advanced glycation end products. Circ Res. 2008;102:905–13. 18323529 10.1161/CIRCRESAHA.107.165308

[27] McMullen MR Pritchard MT Wang Q Millward CA Croniger CM Nagy LE. Early growth response-1 transcription factor is essential for ethanol-induced fatty liver injury in mice. Gastroenterology. 2005;128:2066–76. 15940638 10.1053/j.gastro.2005.02.065 PMC1959407

[28] Body-Malapel M Djouina M Waxin C. The RAGE signaling pathway is involved in intestinal inflammation and represents a promising therapeutic target for inflammatory bowel diseases. Mucosal Immunol. 2019;12:468–78. 30542111 10.1038/s41385-018-0119-z

[29] Shou X Wang Y Zhang X. Network pharmacology and molecular docking analysis on molecular mechanism of Qingzi Zhitong decoction in the treatment of ulcerative colitis. Front Pharmacol. 2022;13:727608. 35237152 10.3389/fphar.2022.727608 PMC8883437

[30] Duan ZL Wang YJ Lu ZH. Wumei Wan attenuates angiogenesis and inflammation by modulating RAGE signaling pathway in IBD: network pharmacology analysis and experimental evidence. Phytomedicine. 2023;111:154658. 36706698 10.1016/j.phymed.2023.154658

[31] Kordjazy N Haj-Mirzaian A Haj-Mirzaian A. Role of toll-like receptors in inflammatory bowel disease. Pharmacol Res. 2018;129:204–15. 29155256 10.1016/j.phrs.2017.11.017

[32] Lu Y Li X Liu S Zhang Y Zhang D. Toll-like receptors and inflammatory bowel disease. Front Immunol. 2018;9:72. 29441063 10.3389/fimmu.2018.00072 PMC5797585

[33] Cui Y Hu J Li Y. Integrated network pharmacology, molecular docking and animal experiment to explore the efficacy and potential mechanism of Baiyu decoction against ulcerative colitis by enema. Drug Des Devel Ther. 2023;17:3453–72. 10.2147/DDDT.S432268 PMC10680469 38024534

[34] Qu Y Li X Xu F. Kaempferol alleviates murine experimental colitis by restoring gut microbiota and inhibiting the LPS-TLR4-NF-κB axis. Front Immunol. 2021;12:679897. 34367139 10.3389/fimmu.2021.679897 PMC8339999

[35] Olaitan Balogun S Sabino Damazo A Pavan E. Evidence for the involvement of cytokines modulation and prokinetic properties in gastric ulcer healing effects of *Helicteres sacarolha* A. St.-Hil. A. Juss. Chem Biodivers. 2022;19:e202200322. 36269048 10.1002/cbdv.202200322

[36] Gang S Bai W Yu H A G Wang Z. *Dracocephalum moldavica* L. extract alleviates experimental colitis in rats by modulating gut microbiome and inflammatory pathways. Mol Med Rep. 2023;28:228. 37859616 10.3892/mmr.2023.13115

[37] Xu X Zhang J Chen L. *Alhagi pseudalhagi* extract exerts protective effects against intestinal inflammation in ulcerative colitis by affecting TLR(4)-dependent NF-κB signaling pathways. Front Pharmacol. 2021;12:764602. 34803708 10.3389/fphar.2021.764602 PMC8600043

[38] Gu S Xue Y Zhang Y. An investigation of the mechanism of rapid relief of ulcerative colitis induced by five-flavor sophora flavescens enteric-coated capsules based on network pharmacology. Comb Chem High Throughput Screen. 2020;23:239–52. 32116186 10.2174/1386207323666200302121711 PMC7475943

[39] Mahmoud TN El-Maadawy WH Kandil ZA Khalil H El-Fiky NM El Alfy TSMA. Canna x generalis L.H. Bailey rhizome extract ameliorates dextran sulfate sodium-induced colitis via modulating intestinal mucosal dysfunction, oxidative stress, inflammation, and TLR4/ NF-ҡB and NLRP3 inflammasome pathways. J Ethnopharmacol. 2021;269:113670. 10.1016/j.jep.2020.113670 33301917

[40] Musso A Dentelli P Carlino A. Signal transducers and activators of transcription 3 signaling pathway: an essential mediator of inflammatory bowel disease and other forms of intestinal inflammation. Inflamm Bowel Dis. 2005;11:91–8. 15677901 10.1097/00054725-200502000-00001

[41] Xu L Zhang J Wang Y Zhang Z Wang F Tang X. Uncovering the mechanism of Ge-Gen-Qin-Lian decoction for treating ulcerative colitis based on network pharmacology and molecular docking verification. Biosci Rep. 2021;41:BSR20203565. 33409535 10.1042/BSR20203565 PMC7876598

[42] Abo H Flannigan KL Geem D Ngo VL Harusato A Denning TL. Combined IL-2 immunocomplex and anti-IL-5 mAb treatment expands Foxp3(+) Treg cells in the absence of eosinophilia and ameliorates experimental colitis. Front Immunol. 2019;10:459. 30930900 10.3389/fimmu.2019.00459 PMC6428029

[43] Wang Z Vaughan TY Zhu W. Gab2 and Gab3 redundantly suppress colitis by modulating macrophage and CD8(+) T-cell activation. Front Immunol. 2019;10:486. 30936879 10.3389/fimmu.2019.00486 PMC6431666

[44] Wei M Li H Li Q. Based on network pharmacology to explore the molecular targets and mechanisms of Gegen Qinlian decoction for the treatment of ulcerative colitis. Biomed Res Int. 2020;2020:5217405. 33299870 10.1155/2020/5217405 PMC7710413

[45] Durant L Watford WT Ramos HL. Diverse targets of the transcription factor STAT3 contribute to T cell pathogenicity and homeostasis. Immunity. 2010;32:605–15. 20493732 10.1016/j.immuni.2010.05.003 PMC3148263

[46] Tan YY Ding Y Zheng X. Ding’s herbal enema treats dextran sulfate sodium-induced colitis in mice by regulating the gut microbiota and maintaining the Treg/Th17 cell balance. Exp Ther Med. 2021;22:1368. 34659514 10.3892/etm.2021.10802 PMC8515548

[47] Ding K Tan YY Ding Y. β-Sitosterol improves experimental colitis in mice with a target against pathogenic bacteria. J Cell Biochem. 2019;120:5687–94. 30548286 10.1002/jcb.27853

[48] Ek WE Karlsson T Höglund J Rask-Andersen M Johansson A. Causal effects of inflammatory protein biomarkers on inflammatory diseases. Sci Adv. 2021;7:eabl4359. 34878845 10.1126/sciadv.abl4359 PMC8654293

